# Adherence and retention on antiretroviral therapy in a public-private partnership program in Nigeria

**DOI:** 10.7448/IAS.15.6.18096

**Published:** 2012-11-11

**Authors:** K Torpey, O Ogbanufe, F Babatunde, O Mosuro, A Fajola, H Khamofu, S Odafe, A Barinaadaa

**Affiliations:** 1 FHI 360, Prevention, Care and Treatment, Garki, Nigeria; 2 FHI 360, Prevention, Care and Treatment, Abuja, Nigeria; 3 SPDC, Corporate Community Health, Warri, Nigeria; 4 FHI 360, Corporate Community Health, Port Harcourt, Nigeria; 5 FHI 360, Prevention, Care and Treatment, Warri, Nigeria

## Abstract

Initiation of HIV-positive patients on antiretroviral therapy (ART) in Nigeria was restricted to secondary and tertiary level hospitals due to weak health systems in primary health centres (PHCs). Shell Petroleum Development Company (SDPC) Nigeria and FHI 360 using a systems strengthening approach, piloted ART enrolment in a PHC in south-eastern Nigeria. This study sought to evaluate patients’ adherence and mortality on ART, and associated risk factors. We reviewed clinic records of adult patients initiating ART between January 2007 and December 2009. Adherence was calculated as the number of days of medication dispensed as a percentage of total number of days evaluated. Outcome measures were probability of being alive and retained in care at 12 and 24 months on ART. Competing risks regression models were used to assess potential predictors associated with mortality. Total of 196 patients (64.8% males) were initiated on ART. Patients’ median age was 35 years (IQR 30–44); median CD4 at initiation was 132 cells/mm^3^ (IQR 82–212), Patients in WHO stage III and IV constituted 73 (37.6%) and 83 (42.8%) respectively. Majority (108 [55.1%]) of patients had adherence rates >95%. Adherence levels ranged: 70–85%, 50–65% and <50% in 29 (14.8%), 30 (15.3%) and 29 (14.8%) of patients respectively. Nucleoside backbone use were AZT/3TC (69.4%) d4T/3TC (28.6%) and TDF/FTC (2%). At 12 months of follow up, 80.6% (158) were alive and on ART, mortality accounted for 12.8% (25), 11 (5.6%) were LTFU and 2 (1.1%) transferred out. At 24 months on ART survival decreased to 64.3% (126), 20.4% (40) died, 9.2% (18) were LTFU and 12 (6.1%) transferred out. Competing risks regression models revealed that patients’ factors significantly associated with mortality include: bedridden patients (HR=3.6 [95% CI: 1.11–11.45], p=0.03, referent: working), <50% adherence levels (HR=27.7 [95% CI: 8.55–89.47], p<0.0001, referent: >95% adherence level). In conclusion, majority of attrition was due to mortality. Poor adherence was associated with 27 times higher risk of death compared with patients with >95% adherence. Mortality is likely to reduce by establishing a more robust adherence counselling process.

